# The phase of pre-stimulus alpha oscillations influences the visual perception of stimulus timing

**DOI:** 10.1016/j.neuroimage.2016.02.065

**Published:** 2016-06

**Authors:** Alex Milton, Christopher W. Pleydell-Pearce

**Affiliations:** School of Experimental Psychology, University of Bristol, 12a Priory Road, Bristol BS8 1TU, United Kingdom

## Abstract

This study examined the influence of pre-stimulus alpha phase and attention on whether two visual stimuli occurring closely in time were perceived as simultaneous or asynchronous. The results demonstrated that certain phases of alpha in the period immediately preceding stimulus onset were associated with a higher proportion of stimuli judged to be asynchronous. Furthermore, this effect was shown to occur independently of both visuo-spatial attention and alpha amplitude. The findings are compatible with proposals that alpha phase reflects cyclic shifts in neuronal excitability. Importantly, however, the results further suggest that fluctuations in neuronal excitability can create a periodicity in neuronal transfer that can have functional consequences that are decoupled from changes in alpha amplitude. This study therefore provides evidence that perceptual processes fluctuate periodically although it remains uncertain whether this implies the discrete temporal framing of perception.

## Highlights

• Pre-stimulus alpha phase influences the perceived timing of two visual stimuli.
• Phase effects were independent of both spatial attention and alpha amplitude.
• The results are compatible with the neural excitability hypothesis of alpha phase.

## Introduction

The study of pre-stimulus oscillatory EEG activity has been instrumental in demonstrating that variation in perceptual processing is related to changes in the ongoing state of neuronal activity. Spatial cueing paradigms have shown that attention selectively alters pre-stimulus activity within the alpha band (8–13 Hz) with alpha amplitude reduction correlated with improved performance (e.g. Thut et al., 2006) and amplitude increments associated with impaired performance (e.g. Kelly et al., 2006). Moreover, these effects are localized over task-relevant areas (e.g. Worden et al., 2000). Given specific temporal expectations, the time-course of these changes in alpha amplitude will vary so that they correspond with anticipated stimulus presentation (Rohenkohl and Nobre, 2011). Accordingly, the modulation of ongoing neuronal activity that is indexed by changes in alpha is thought to be important in the deployment of attention over time.

While amplitude changes typically occur on the scale of seconds or hundreds of milliseconds, the faster fluctuation of neuronal activity reflected by changes in phase are also thought to be important. When stimulus occurrence is temporally unpredictable, the detection rate of near-threshold (Busch et al., 2009) and backward-masked (Mathewson et al., 2009) visual stimuli has been shown to vary according to the phase of coincident alpha activity, as has the detection of auditory stimuli (Rice and Hagstrom, 1989). When stimulus presentation is predictable, preferential alignment of alpha phase to stimulus onset is observed (e.g. Barry et al., 2004) that has been shown to improve perception (Mathewson et al., 2012, Spaak et al., 2014). This selective alignment of phase coheres with the idea that particular phases correspond to temporal windows of increased excitability that improve processing (Lindsley, 1952). Furthermore, studies inducing changes in alpha phase using rhythmic transcranial magnetic stimulation (Dugué et al., 2011) and oscillating transcranial direct current stimulation (Neuling et al., 2012) have demonstrated that phase plays an important role in influencing subsequent sensory processing. However, while these detection studies show that the tendency for singular events to be detected versus missed is dependent upon the phase of coincident alpha activity, it remains unclear whether the fluctuation of phase over time also influences the perceived timing of stimuli. Support for this assertion was provided by Varela et al. (1981) when they reported that the likelihood participants would perceive two asynchronous stimuli as occurring simultaneously or asynchronously depended upon the phase of alpha with which the stimuli were coincident, even though the temporal asynchrony of the stimuli was constant. The results suggested that judgements of relative timing were in part influenced by the underlying state of neuronal activity, and that processes involved in the temporal integration or segregation of separate events might bear some relation to ongoing changes in alpha phase. A more modest but nonetheless positive finding was reported in a follow-up study (Gho and Varela, 1988). However, subsequent attempts to replicate these findings have reportedly been unsuccessful (see VanRullen and Koch, 2003, pg. 209).

This paper revisits the simultaneity paradigm in order to explore whether the theoretically important relationship between alpha phase and temporal perception could be replicated. As the effect has proved difficult to replicate, we considered the potentially important role of attention and alpha amplitude in mediating effects of alpha phase. On account of attention-related changes in alpha amplitude, modern theories of alpha activity have suggested that it plays a functional role in cortical inhibition (e.g. Klimesch et al., 2007, Mathewson et al., 2009, Jensen and Mazaheri, 2010). Phase has been proposed to reflect the rapid time-scale modulation (or pulsing) of this inhibition that therefore engenders periods of improved and inhibited processing that are more pronounced at higher amplitudes (Klimesch et al., 2007, Mathewson et al., 2009, Jensen et al., 2012). This suggests that effects of alpha phase may be more detectable during increased levels of alpha amplitude associated with the absence of attention. This study therefore employed a lateralized task where attention was cued toward or away from stimuli requiring a judgement of simultaneity. This allowed us to assess directly the relationship between alpha phase and amplitude on simultaneity judgements at electrodes where amplitude changes are demonstrably correlated with attention.

## Material and methods

### Participants

24 participants (5 male; age range: 19–29) took part in the experiment in return for £20 reimbursement. All had normal or corrected-to-normal vision and gave written informed consent in accordance with the University of Bristol Faculty of Science Human Research Committee. Data from four participants were excluded from further analysis on the basis of excessive eye movements during trials (> 20% of trials). This left 20 participants (2 male; age range 19–28) in the final analysis.

### Stimuli & procedures

**Fig. 1:**
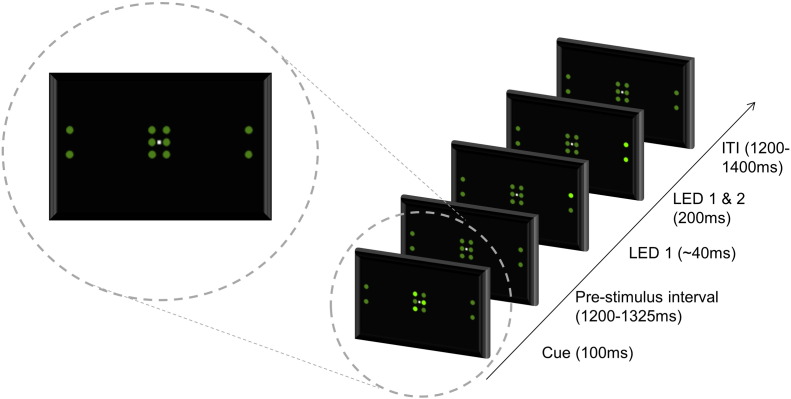
The LED sequence and timings for an individual trial. This trial represents a valid cue for a right visual field event with asynchronous onset of LEDs.

All stimuli were presented using a custom display box consisting of 6 central LEDs used for spatial cueing and 4 LEDs, 2 lateralized either side, for presentation of targets (Fig. 1). Fixation was a white dot in the centre of the display and was set at eye-level. The display was positioned 50 cm from the chin rest. All LEDs were 5 mm in diameter and lateralized LEDs were 8.58° from central fixation (this is within the range of eccentricities used in similar studies: 4.1°, Romei et al., 2010; 6.5°, Sauseng et al., 2005; 26.5°, Thut et al., 2006). All timings and response collections were written and controlled using the psychophysics toolbox in MatLab (Brainard, 1997).

The experiment asked participants to make a simple judgement concerning the respective onset of 2 lateralised LEDs. Participants always maintained central fixation but were asked to orient their attention covertly to the pair of lateralized LEDs indicated by the cue (Fig. 1). Each trial started with a centrally-presented spatial cue that lasted 100 ms followed by a variable pre-stimulus interval of 1200–1325 ms. For asynchronous trials, there was a stimulus onset asynchrony (SOA) between the onset of the first illuminated LED (top- versus bottom-leading trials were fully counterbalanced) and the onset of the second LED. SOAs were determined via individual thresholds (see later), but after the SOA both target LEDs remained illuminated for 200 ms before undergoing coincident offset. Due to the SOA, the leading LED persisted for a longer duration than the second. For durations up to 100–150 ms, increased duration of a stimulus is associated with an increase in its perceived brightness (Osaka, 1977, Rieiro et al., 2012). The 200 ms co-persistence of both LEDs before simultaneous offset meant there would be no differences in perceived brightness that might have influenced the judgement. For simultaneous trials, both LEDs were coterminous and lasted 200 ms. The trial would not terminate until a response was given. Between trials there was a randomised variable duration of 1200–1400 ms.

A total of 680 trials were undertaken across 5 blocks of 136 trials. Each block was further divided into 4 mini-blocks of 34 trials between which were 20 s enforced breaks. Breaks between blocks were self-paced. There were 4 trial types: correctly-cued asynchronous trials (420 trials), incorrectly-cued asynchronous trials (140 trials), correctly-cued simultaneous trials (90 trials) and incorrectly-cued simultaneous trials (30 trials). In order to ensure that participants oriented attention in line with the cue, correctly-cued trials constituted 75% of all trials which is commensurate with previous studies showing pre-stimulus alpha lateralization in response to spatial cues (75%, Sauseng et al., 2005; 66%, Thut et al., 2006). Simultaneous trials were included to give information on false alarm rates and sensitivity but were not intended for EEG analysis. The ratio of trial types was consistent across mini-blocks and blocks. The side of presentation (left versus right) and which LED occurred first (top versus bottom) were fully counterbalanced across trial types and experimental blocks. SOAs for asynchronous stimuli were subject to a threshold procedure to keep the proportion of asynchronous judgements around .5 in line with the original study design (Varela et al., 1981). SOA thresholds for correctly and incorrectly-cued trials were determined separately via a running staircase procedure (used previously by Busch and VanRullen, 2010). After initial approximation of threshold during practice trials (*n* = 40), SOAs were subsequently reduced by 5 ms if the previous response was correct and increased by 5 ms if it was incorrect.

Responses were given on a handheld 9-digit number pad. In order to avoid a lateralised motor response in the EEG, participants gave bi-manual responses (1 & 3, asynchronous; 4 & 6, simultaneous). Before the experiment started, participants completed two short tasks. First they completed 30 trials where they were asked to saccade from central fixation to the lateralized LEDs (15 trials to each side). This task allowed the individual characterisation of saccadic eye-movements upon which artefact exclusion measures could be based (see *EEG acquisition and analysis* below). Second, participants completed 2 practice blocks of 40 trials each. These were identical to the main experimental blocks except that no simultaneous trials were included. Initial SOAs for both attention conditions were set at 50 ms, but the SOA values at the end of the practice block were used as the starting values in the main experiment.

### EEG acquisition and analysis

EEG data were recorded from 30 Ag/AgCl electrodes located in accordance with the International 10–20 system. All EEG electrodes were referenced to two linked electrodes on the left and right mastoid. Vertical and Horizontal electro-oculogram recordings were taken from electrodes placed above and below the right eye (VEOG) and both outer canthi (HEOG). Electrode impedances were kept < 10 kΩ. EEG and EOG data were acquired using a Contact Precision amplifier with a highpass filter of 0.03 Hz and a lowpass at 200 Hz. Data in all channels were sampled at 1000 Hz and then downsampled and saved at 500 Hz. All subsequent data screening and processing were undertaken in MatLab (MathWorks) using custom scripts and functions from the signal processing toolbox. Offline, data were filtered forwards and backwards using a first order Butterworth high pass filter with a cut-off at .1 Hz and a fourth order Butterworth low pass filter with a cut-off at 30 Hz. This resulted in a high pass filter with a half-amplitude cut-off of .1 Hz and a roll-off of 12 dB per octave and a low pass filter with a half-amplitude cut-off of 30 Hz and a roll-off of 48 dB per octave.

In order to detect and remove trials with saccades, the average absolute magnitude of left and right saccades was determined for each participant on the basis of the initial saccadic task. A threshold magnitude was determined for each participant that corresponded to 20% of their average saccadic amplitude. The main data were then segmented into trial epochs defined as − 1350 ms to 250 ms post onset of the first target LED and they were scanned for activity in HEOG. Saccades were identified by running a 200 point (400 ms) analysis window point by point through the trial epochs (suggested by Luck, 2005). The mean voltage value from points 1–100 was subtracted from the mean voltage value across points 101–200, and if the absolute difference exceeded the threshold value then the trial was rejected from further analysis for all electrodes. After 4 participants were removed from further analysis for > 20% of trials contaminated by saccades, a mean of 4% of trials were rejected on this basis for the remaining 20 participants. Blinks clearly represent periods when participants are insensitive to visual stimuli and we employed an artefact rejection procedure because our task involved the judgement of brief intervals between visual stimuli. In order to identify trials contaminated by blinks or similar ocular artefacts, we used a template-matching procedure (defined below). Shorter trial epochs were defined as ± 400 ms around onset of the first target LED, and any epoch contaminated with artefact activity was discarded from analysis for all electrodes (mean of 1.59% trials rejected). Artefact templates were defined for each participant using the vertical electro-oculogram (VEOG). All data in VEOG were first converted to z-scores by subtracting the mean of all time points from the value at each time point and then dividing this value by the standard deviation of all time points. Any time points where z-scores exceeded ± 2 were defined as regions of artefact activity and any trial whose epoch overlapped with this activity was removed from the main analysis for all electrodes. Next, where VEOG z-scores > 2 were identified, the number of time points for which they persisted was calculated. Each region of artefact activity was then given a z-score based on its duration. All artefact regions with a length within ± 2 z-scores were then averaged together to form a template of artefact activity for that participant. Before averaging, these artefact regions were aligned centrally by their maximum value and regions of shorter length were zero-padded so that they shared a common length with longer regions. This same process was performed separately for z-scores <− 2. These templates were then moved through the VEOG data point-by-point and a correlation between the data and the template was calculated. For correlations > 0.7, the starting point of the data at which the template was overlaid was noted as well as the correlation value. As multiple adjacent time points would register *r* values > 0.7, the time point with the highest *r* value within such regions was selected as the best time region incorporating artefact activity. The maximum z-score for each region was then established and the first points preceding and following that maximum value at which z-scores < .5 were recorded (or >−.5 for negative artefacts). These were taken as the points at which activity deviated from normal and were below the initial criterion of z-score >± 2 used to detect artefact activity. This meant that longer artefact windows could be established with the template procedure. Again, any trial epoch that overlapped with these artefact windows was excluded from analysis for all electrodes. Finally, for each electrode, remaining trial epochs where activity exceeded ± 75 uV were also removed from analysis for that electrode (e.g. Busch et al., 2009). The average number of remaining trials at each electrode was 640.70, *SD* = 22.84 (lowest number of observations = 585).

For the main analysis, only data from asynchronous trials was analysed, and, because stimuli were lateralized, midline electrodes were not analysed. Trial epochs were defined as 201 point (402 ms) windows extending from − 150 points (− 300 ms) before onset of the first target LED to + 50 points (100 ms) after its onset. Epochs were Tukey tapered with 50 point cosines. This epoch allowed analysis of untapered pre-stimulus EEG activity from − 200 ms up to and including stimulus onset similar to approaches employed by other studies investigating the influence of pre-stimulus alpha on visual perception (e.g. Mathewson et al., 2009). Alpha amplitude was computed using the fast Fourier transform (FFT) on the 201 point, tapered window defined above that was zero-padded to 512 points. This gave a frequency resolution of .977 Hz. Alpha was analysed for coefficients 7.813–12.695 Hz. Analysis of the attentional modulation of pre-stimulus alpha amplitude was computed across both correctly and incorrectly-cued trials as this window preceded subsequent cue validity, excluding the tapered section. For each electrode, trials were grouped by whether the cue allocated attention to contralateral (attended) or ipsilateral (unattended) visual space. The effect of attention on alpha amplitude at each electrode was compared using a paired-samples t-test. The problem of multiple comparisons was controlled using cluster-based permutation analysis (Maris and Oostenveld, 2007, Nichols and Holmes, 2001). 10,000 Monte Carlo permutations were run with an initial entry-threshold of 0.05 for permutation clusters.

Instantaneous phase of pre-stimulus alpha was computed for each electrode using a Gabor filter (an approach previously used by Hanslmayr et al., 2013) on the tapered epochs described above. The Gabor filter was specified by each participant's individual alpha frequency (IAF). IAF was calculated separately for frontal (FP1/2, F3/4, F7/8, FC3/4, FT7/8, C3/4, T7/8, FZ, FCZ, CZ) and posterior (CP3/4 TP7/8, P3/4, P7/8, O1/2, PZ, POZ, OZ) electrodes. Fourier coefficients in the alpha band (7.813–12.695 Hz) for the 200 ms pre-stimulus period were calculated for each electrode and then averaged across frontal and posterior electrodes. The coefficient with the largest average magnitude was defined as the IAF and this value was used to set the Gabor filter for participating electrodes. As our lateralized paradigm was explicitly designed to explore the interaction between alpha phase and attention, phase analysis was restricted to electrodes showing a pre-stimulus attentional modulation of alpha amplitude. For each participant and each electrode within relevant electrodes, trials were separated by perceptual outcome (asynchronous versus simultaneous) and the mean phase and resultant vector length was calculated at every time point from − 200 ms to stimulus onset (Fs = 500 Hz, 101 time points). At this stage data were collapsed across attention conditions. In order to ensure an equal number of phase angles contributed to the circular mean for each outcome, a subsampling procedure was used where a random selection of trials (without replacement) equal to the total in the condition with fewer trials was used for the condition with greater trials. For each participant, a second-order circular mean that accounts for mean phase values and resultant vector lengths (Zar, 1999) was calculated for each time point across the electrodes of interest in order to give a cluster value. These second-order means were then pooled across participants for the two perceptual outcomes separately so that phase differences could be assessed from − 200 ms to stimulus onset using a second-order non-parametric paired-samples test (Zar, 1999) corrected for multiple comparisons using permutation-based cluster analysis. As a nonparametric test, no underlying assumptions about the distribution are made and the test is appropriate for assessing phase differences in paired-samples data.

As there were no known appropriate statistics for assessing multi-factorial paired-samples circular data, phase and attention interactions were investigated using the proportion of correctly perceived asynchronous trials as the dependent variable. For this analysis, only trials where targets appeared in the contralateral visual field to the electrodes analysed were included, and the attention condition denoted whether the trial was correctly or incorrectly-cued. For this analysis of the proportion of correctly perceived asynchronous trials, phase was defined at the precise moment of stimulus onset (as also performed by Varela et al., 1981; see also Cravo et al., 2015). Phase classification involved two bins chosen to bisect phase angles equally between the across-participant mean phase associated with each perceptual response in the initial phase analysis (see Fig. 3). Accordingly, a 2 × 2 paired-samples ANOVA investigated the effect of Attention and Alpha phase on the proportion of correctly perceived asynchronous trials. Proportion data for the four conditions was calculated separately for each participant and relevant electrode and then averaged across cluster electrodes.

Where effect sizes are included, partial-eta squared is reported for ANOVAs and Cohen's *d*_av_ with Hedge's *g*_av_ correction is reported for t-tests as recommended by Lakens (2013).

## Results

### Behavioural data

#### Performance

The average hit rate of asynchronous stimuli correctly perceived as asynchronous was .496 (*SD* = .023) revealing that the running staircase procedure successfully kept performance close to threshold. The average hit rate for correctly-cued versus incorrectly-cued trials was .497 (*SD* = .032) and .494 (*SD* = .010), respectively and this difference was not statistically significant (*t*(19) = .468, *p* = .645, Hedge's *g*_av_ = .068). This indicates that the separate threshold procedure for the two conditions performed as intended. The average SOA across both correctly-cued and incorrectly-cued trials was 39 ms (*SD* = 12 ms). The overall false alarm rate was low (*M* = .043, *SD* = .023; correctly-cued: *M* = .041, *SD* = .021; incorrectly-cued: *M* = .045, *SD* = .029) suggesting that participants could adequately perform the task, and the overall *d*′ value supports this conclusion (*M* = 1.778, *SD* = .260). There was no difference in *d*′ (*t*(19) = .943, *p* = .358, Hedge's *g*_av_ = .157) or the criterion (*t*(19) = .534, *p* = .599, Hedge's *g*_av_ = .058) for correctly-cued versus incorrectly-cued trials which suggests the respective threshold procedure for both trials types performed as intended and that participants did not adopt differing response strategies for the two trial types.

#### Attentional manipulation

In order to ensure that participants did orient attention in line with the pre-stimulus cue, two behavioural measures were checked. First, the response times associated with correctly-cued versus incorrectly-cued targets were compared. A paired-samples t-test showed that response times were faster to correctly-cued targets (*M* = 523 ms, *SD* = 123 ms) than incorrectly-cued targets (*M* = 583 ms, *SD* = 141 ms), *t*(19) = − 5.164, *p* = 5.526 × 10^− 5^, Hedge's *g*_av_ = .461), and this suggests that attention was allocated in line with the pre-stimulus cue.

Second, as the online thresholding of SOA values was undertaken separately for correctly-cued versus incorrectly-cued targets, it was also possible to analyse whether there was a difference between the average SOA threshold for the two conditions. A paired-samples t-test revealed statistically lower SOAs for correctly-cued trials (*M* = 37 ms, *SD* = 12 ms) than incorrectly-cued trials (*M* = 41 ms, *SD* = 12 ms) (*t*(19) = − 5.400, *p* = 3.280 × 10^− 5^, Hedge's *g*_av_ = .207). The lower SOA threshold for the correctly-cued condition provides further evidence that participants were allocating attention in line with the cue.

### Electrophysiological data

#### Attentional modulation of alpha amplitude

**Fig. 2:**
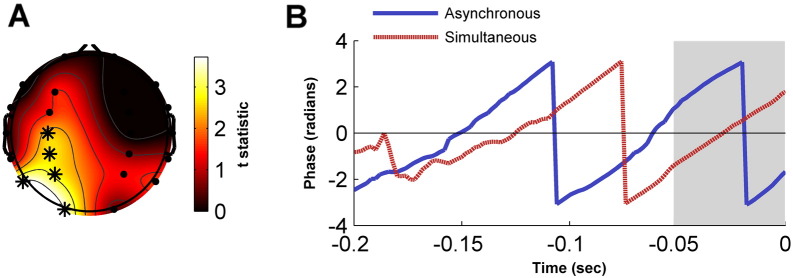
(A) Topographic plot of attentional differences in pre-stimulus alpha amplitude as assessed by paired samples t-test. Asterisks denote electrodes displaying statistically significant attentional modulation of alpha amplitude. (B) Grand average alpha phase at the cluster-level for the two perceptual outcomes in the pre-stimulus period. The grey shaded area depicts the time region where phase was statistically different between conditions. As the results depict the average phase across participants, the absence of phase consistency (e.g. − 0.2 to − 0.15 s) indicates time regions at which phase relationships were not consistent across participants or electrodes of interest.

The influence of attention on alpha amplitude in the pre-stimulus period was investigated using permutation-based cluster analysis. This identified a cluster of five left hemisphere electrodes (C3, CP3, P3, P7 & O1) that exhibited lower alpha amplitude when the cue oriented attention to the contralateral visual field than when attention was oriented to the ipsilateral visual field (Monte Carlo *p* = .009) (see Fig. 2A).

As a conservative confirmation that the attentional modulation of pre-stimulus alpha was indeed lateralised to the left hemisphere and was not instead influenced by hemispheric differences in absolute alpha amplitude, two further analyses were undertaken. For each hemisphere separately, the influence of the attention condition on the five posterior electrodes identified (or their right hemisphere homotopic equivalents) was investigated by means of a 2 factor (5 × 2) paired-samples ANOVA. For the left hemisphere, there was a main effect of attention (*F*(1,19) = 12.483, *p* = .002, η_p_^2^ = .396) but the interaction was not statistically significant (*F*(2.30,43.73) = 1.560, *p* = .219, η_p_^2^ = .076). For the right hemisphere, there was no main effect of attention on alpha amplitude (*F*(1,19) = 1.096, *p* = .308, η_p_^2^ = .055) and no interaction (*F*(1.81,34.46) = 1.329, *p* = .276, η_p_^2^ = .065). The attention-mediated alpha suppression effect therefore appears to be left localized.

#### Alpha phase

**Fig. 3:**
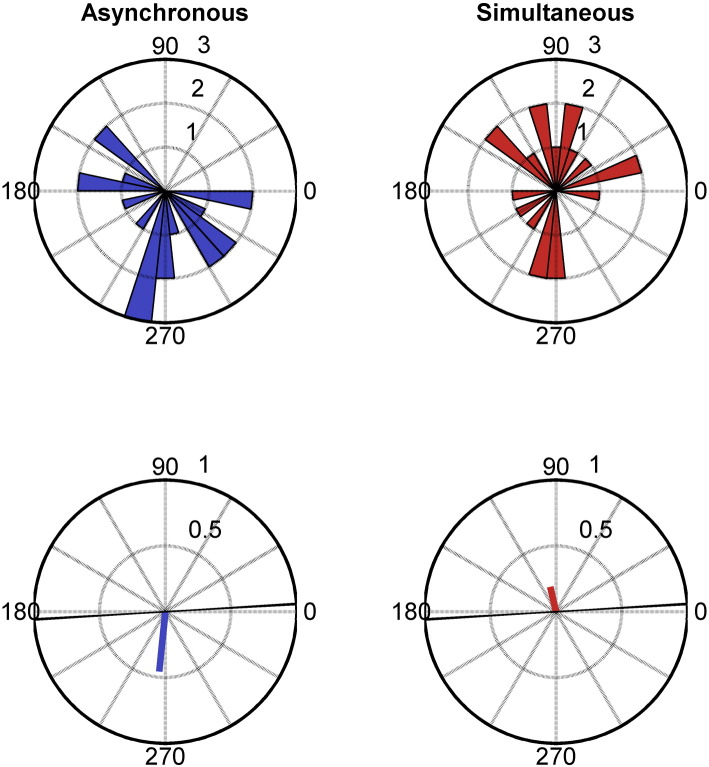
Cluster-level alpha phase distributions (top) and mean phase angle (bottom) for the two perceptual outcomes at stimulus onset (*R*_*19*_′ = 1.325, *p* < .01). The black line in the bottom images reflects a circular bisection between the 2 across-participant means that was used for further analysis described in Results section. 270°= negative peak.

As this paper is interested in investigating whether any effects of alpha phase on judgements of simultaneity are contingent upon attention-dependent levels of alpha amplitude, the effect of alpha phase on simultaneity were investigated for the cluster of electrodes showing an attentional modulation of alpha amplitude. For each participant, trials were separated by perceptual outcome and the second-order mean phase and resultant vector length was calculated across cluster electrodes at every time point from − 200 ms to stimulus onset. Differences in across-participant phase distributions for the two perceptual outcomes were assessed from − 200 ms to stimulus onset using a second-order non-parametric paired-samples test (Zar, 1999) corrected for multiple comparisons using permutation-based cluster analysis. This revealed that the two perceptual outcomes were associated with statistically different phases for a time period of − 52 ms to stimulus onset (Monte Carlo *p* = .042) (see Fig. 2B). The across-participant phase distribution and mean phase angle are displayed for stimulus onset in Fig. 3.

Next, the influence of attention and alpha phase on the proportion of correctly perceived asynchronous trials was investigated. Phase angles at stimulus onset were binned according to a circular bisection based upon the phase angles midway between the circular means for each response (see Fig. 3). A 2 × 2 paired-samples ANOVA investigated the effect of Attention and Alpha phase on the proportion of correctly perceived asynchronous trials. Unsurprisingly, there was a main effect of alpha phase (*F*(1,19) = 5.135, *p* = .035, η_p_^2^ = .213) which revealed that the phase bin at stimulus onset defined by asynchronous trials was associated with a greater proportion of correctly identified asynchronous trials (*M* = .513, corrected *SE* = .010) than the phase bin defined by simultaneous trials (*M* = .491, corrected *SE* = .010). Interestingly, there was neither a main effect of attention (*F*(1,19) = .616, *p* = .442, η_p_^2^ = .013) nor an interaction between attention and phase (*F*(1,19) = .633, *p* = .436, η_p_^2^ = .032). As the pulsed-inhibition hypothesis predicts that alpha phase effects should be more pronounced at higher amplitudes associated with unattended conditions, this latter finding is contrary to one of the main predictions of the study.

Although attentional modulation of alpha amplitude was demonstrated at the cluster electrodes included in the phase analysis, a further analysis was conducted to check explicitly whether alpha phase effects were independent of pre-stimulus alpha amplitude as well as attention. For each electrode in the cluster, trials were pooled across the attention conditions and then grouped into high versus low alpha amplitude on the basis of a median split. The proportion of asynchronous responses was calculated for four conditions on the basis of Phase bin (defined by asynchronous trials versus simultaneous trials as described above) and Amplitude (low versus high). For each participant, data were averaged across electrodes showing an attentional modulation of alpha amplitude in the cluster analysis. A 2 × 2 paired-samples ANOVA investigated whether alpha phase and alpha amplitude influenced the proportion of correctly perceived responses in the simultaneity paradigm. As already reported, there was a main effect of alpha phase (*F*(1,19) = 6.021, *p* = .024, η_p_^2^ = .241), however there was no main effect of alpha amplitude (*F*(1,19) = 1.308, *p* = .267, η_p_^2^ = .064) and no interaction between amplitude and phase (*F*(1,19) = .192, *p* = .666, η_p_^2^ = .010) on the proportion of asynchronous responses. This analysis therefore clarifies that alpha phase effects on the perception of simultaneity were independent of pre-stimulus attention and pre-stimulus alpha amplitude.

As the classification of phase bins used for the above analysis (see Fig. 3) was very close to the original division of phase polarity (positive versus negative alpha peaks) employed in the original study by Varela et al. (1981), a further analysis of phase polarity on the proportion of asynchronous responses was performed using this distinction in order to demonstrate a robust replication of the prior finding. The effect of alpha polarity at stimulus onset was statistically significant (*t*(19) = 2.103, *p* = .049, Hedge's *g*_av_ = .197) demonstrating a higher proportion of asynchronous responses associated with negative polarity (*M* = .512, corrected *SE* = .010) than positive polarity (*M* = .491, corrected *SE* = .010). Similarly, the same proportion data analysis conducted for alpha polarity at stimulus onset as defined using a 10 Hz Gabor filter rather than IAF also revealed a statistically significant effect (*t*(19) = 2.295, *p* = .033, Hedge's *g*_av_ = .271). Again, there was a higher proportion of asynchronous responses associated with negative polarity (*M* = .516, corrected *SE* = .013) than positive polarity (*M* = .487, corrected *SE* = .013).

For all asynchronous trials contributing to the phase analysis reported above, differences in evoked activity for the two perceptual responses were checked at the cluster-average level. ERPs (baselined from − 200 ms to stimulus onset) for asynchronous versus simultaneous responses were compared up to 400 ms post-onset of the first LED using the permutation procedure outlined by Blair and Karniski (1993). This was repeated for both the filtered data and unfiltered data. This revealed no statistically significant differences in evoked activity for the two perceptual responses at any time point within this window (filtered data: *t*_*max*_(19) = 2.150, *p* = .778; unfiltered data: *t*_*max*_(19) = 3.106, *p* = .552). Separate analyses for each cluster electrode also revealed no differences for filtered or unfiltered data. We therefore conclude that the effect of pre-stimulus phase on perceived simultaneity is unlikely to reflect differences in evoked activity that occurred within the post-stimulus taper (1–100 ms) or was smeared backward in time by acausal filtering.

Following the procedure detailed by Busch et al. (2009), we used a Rayleigh test to check that phases at stimulus onset were uniformly distributed across trials for both responses. This was done for each participant at each of the 5 electrodes in the cluster. No *p* value survived Bonferroni correction for comparisons across participants (to be less conservative we did not also correct for multiple comparisons across electrodes). Without correction, one participant showed non-uniformity for electrode C3, and another participant showed non-uniformity for electrodes C3, CP3 and P3. As detailed in Busch et al. (2009), we assessed the probability of observing this finding when trials were drawn from random phases. For each subject and for each electrode, random phases matching the same number of trials in the actual data were drawn. These were assessed using a Rayleigh test, and this procedure was repeated 100,000 times. Using phases from a random distribution, the probability of at least 2 participants having at least one electrode with phases deviating from uniformity (uncorrected Rayleigh test) was .786. The probability of having at least 4 deviations from uniformity across the all participants and electrodes was .743. Our data is therefore thought to be uniform at stimulus onset at the electrodes in the cluster.

#### Secondary analyses

**Fig. 4:**
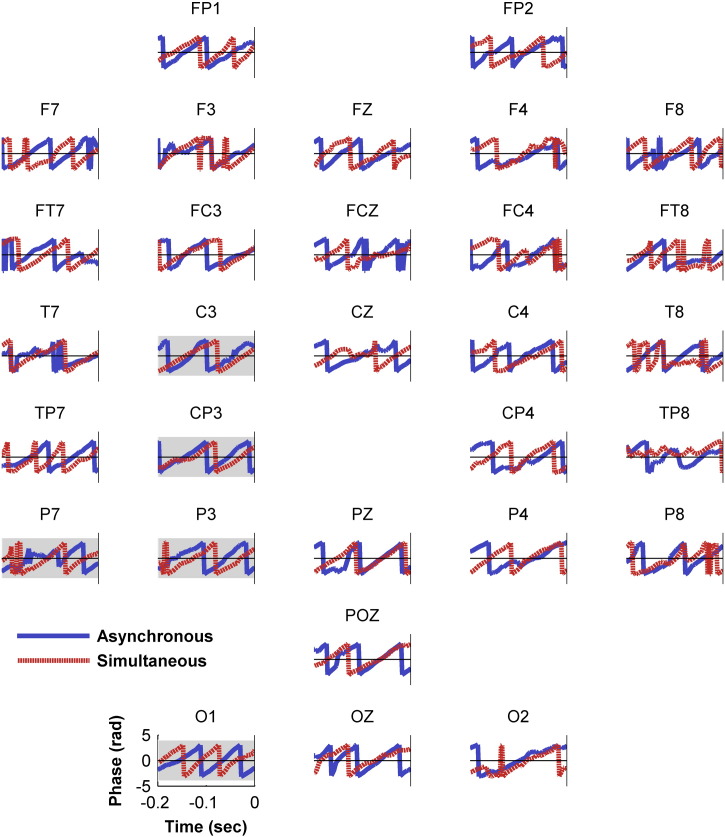
Grand average alpha phase across all electrodes for the two perceptual outcomes in the 200 ms pre-stimulus period. As an unconstrained analysis, no electrodes or time points reached significance, but the grey shaded plots depict the electrodes considered in the original cluster defined by the attentional modulation of alpha amplitude.

As the results showed that the effect of alpha phase on judgements of simultaneity did not interact with attention or alpha amplitude an outstanding question was whether the effect of alpha phase existed beyond those electrodes originally considered for analysis. Accordingly, a further test for differences in alpha phase in the pre-stimulus period (− 200 ms to stimulus onset) was run across all 30 electrodes using the second-order non-parametric paired samples test controlled by cluster-based permutation analysis. This wider analysis revealed no statistically significant phase differences (Monte Carlo *p* = .597; see Fig. 4). This suggests that there were no additional phase effects in other electrodes and also highlights that the phase effects reported in the original analysis are not strong enough to survive the increased noise associated with a larger analysis. The strength of the effect and its restriction to left posterior electrodes demonstrating an attentional modulation of alpha amplitude are considered further in Discussion.

**Fig. 5:**
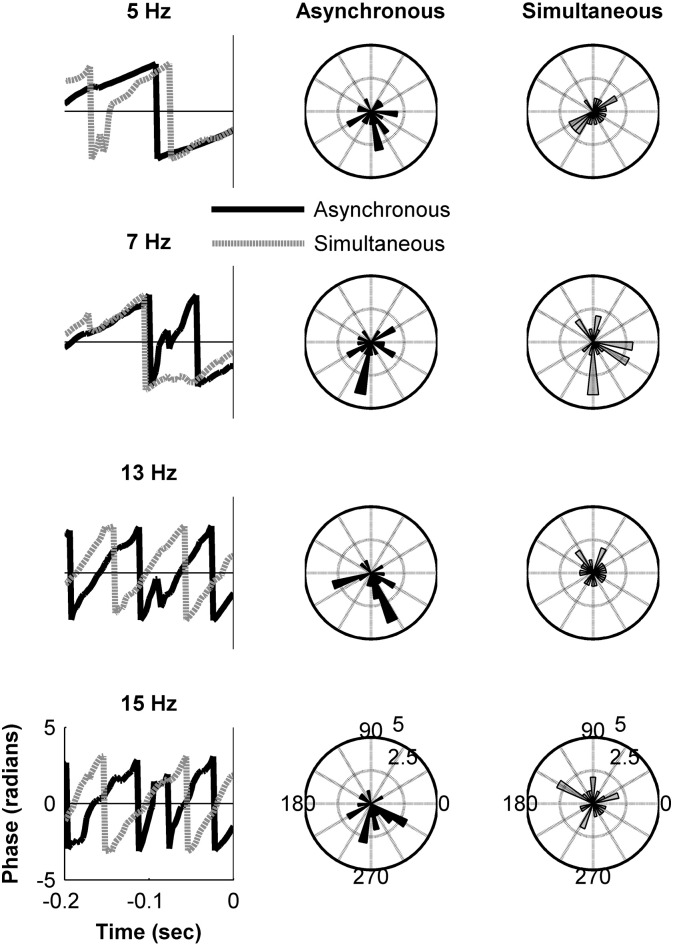
Grand average phase at the cluster-level for the two perceptual outcomes in the pre-stimulus period is depicted for other EEG frequencies alongside cluster-level phase distributions at stimulus onset.

Although our hypotheses concerned the alpha rhythm, a further post-hoc test explored whether phase differences for the two perceptual outcomes in the cluster identified were evident for proximal frequencies in the EEG. Phase was calculated for 5, 7, 13 and 15 Hz using Gabor filters at those frequencies. A similar analysis to that reported at the beginning of Alpha phase section assessed phase differences associated with 5, 7, 13 and 15 Hz for the two perceptual outcomes from − 200 ms to stimulus onset using a second-order non-parametric paired-samples test (Zar, 1999) corrected for multiple comparisons using permutation-based cluster analysis. There was no difference in phase from − 200 ms to stimulus onset for the 5 Hz (Monte Carlo *p* = .174), 7 Hz (Monte Carlo *p* = .279), 13 Hz (Monte Carlo *p* = .195) or 15 Hz (Monte Carlo *p* = .181) (see Fig. 5). This therefore shows that phase differences in the relevant electrodes were constrained to the alpha band and that the phase of proximal frequencies was not associated with differing perceptions of stimulus timing.

## Discussion

This study explored the relationship between pre-stimulus alpha activity and judgements of temporal simultaneity under both attended and unattended conditions. Visuospatial attention was manipulated by cueing attention either toward or away from the lateralized visual field in which subsequent simultaneity targets appeared. Behavioural results demonstrated that correctly-oriented attention was associated with improved reaction times and a lower SOA threshold for the targets. Electrophysiological activity revealed a left-posterior cluster of electrodes that demonstrated an attentional suppression of pre-stimulus alpha amplitude, in line with previous studies (e.g. Sauseng et al., 2005, Thut et al., 2006, Worden et al., 2000). By focusing on this cluster, we demonstrated an influence of pre-stimulus alpha phase on the perception of stimulus simultaneity that was independent of both attention and alpha amplitude. Certain phases of alpha were associated with an increased tendency to correctly perceive the stimuli as asynchronous whereas near-opposite phases were related to a higher proportion of simultaneous judgements. As such we provide renewed evidence for an influence of alpha phase on the visual perception of stimulus timing.

Our findings cohere with numerous reports that pre-stimulus alpha phase influences the efficiency of target processing in terms of reaction time (e.g. Dustman and Beck, 1965, Lansing, 1957) and near-threshold detection (e.g. Busch et al., 2009, Neuling et al., 2012). It is therefore parsimonious to assume that phase oscillations reflect undulations of neuronal excitability (Buzsáki and Draguhn, 2004, Lindsley, 1952). Interestingly, although our analysis explored any possible differences in phase associated with the two perceptual outcomes, the division of the phase cycle at stimulus onset very closely matches the polarity division (positive versus negative peaks) that was reported in the original Varela finding. Our results show that negative phase at stimulus onset favours asynchronous perception. Unfortunately, it is unclear whether the direction of our polarity finding at stimulus onset directly agrees with those of Varela et al. (1981) as their findings are reported differently across the subsections of their paper. It is also likely that pre-stimulus alpha phase is important not at the precise moment of stimulus onset, but instead at a later stage reflecting either thalamocortical transfer or information arrival within the cortex (for a discussion of conduction time see Dustman and Beck, 1965). Thus, phase near stimulus onset may be critical because it predicts an upstream preferential phase. This hypothesis is difficult to test because phase measurements after stimulus onset are confounded with event-related processing (Ritter and Becker, 2009), and this is why we focused on pre-stimulus activity. However, given the polarity effect at stimulus onset reported in the original study and this replication (see also Rice and Hagstrom, 1989), it is tempting to consider biophysical theories of slow and DC cortical potentials which frequently argue that negative polarity reflects raised excitability within vertically-aligned pyramidal neurons often taken to reflect afferent inputs to layer 1 (Elbert, 1993, Mitzdorf, 1985). Given an alpha frequency of 8–12 Hz, negative phase at stimulus onset predicts negative phase around 90–125 ms post onset which fits neatly with typical P1 and N1 peak latencies observed in visual and auditory paradigms respectively. These component peaks are likely to reflect important stages in early cortical processing and they may be particularly susceptible to differences in pre-stimulus neuronal excitability indexed by alpha phase. However, while these arguments are parsimonious they are inconsistent with our failure to demonstrate an interaction between alpha amplitude and phase. For example, if positive phase is disadvantageous, it would be predicted that higher amplitude positive phase would be even more detrimental, but we found no evidence for this. This is an important null finding because an interaction between phase and amplitude is predicted by the theory that alpha phase reflects pulsed inhibition (e.g. Mathewson et al., 2009). On this account, higher amplitude pulses in phase reflect more pronounced periods of inhibition and relative release where phase effects on behavioural outcomes should be more detectable. It is particularly surprising that we observed no interaction of alpha phase and amplitude given that amplitude in our experiment was demonstrated to be modulated by attention. Thus the task provided optimum circumstances to test the pulsed-inhibition hypothesis of alpha phase. The lack of an interaction suggests that fluctuations in alpha phase can have functional consequences that are decoupled from changes in alpha amplitude.

The phase effect demonstrated in this simultaneity paradigm may stem from a different functional characteristic of the alpha phase cycle to the effect of alpha phase highlighted by detection paradigms. Detection paradigms have shown that the phase of pre-stimulus alpha can influence the perceptual fate of a transient near-threshold stimulus (e.g. Busch et al., 2009, Mathewson et al., 2009, Rice and Hagstrom, 1989), which is consistent with the hypothesis that phase reflects fast timescale shifts in neuronal excitability or the level of inhibition. The momentary level of this excitability, influenced by both phase and amplitude, will affect stimulus processing for near-threshold stimuli whose detection will be particularly susceptible to the coincident state of brain activity (Mathewson et al., 2009). In contrast, the temporal paradigm in our study and that of others (Varela et al., 1981, Gho and Varela, 1988) suggests that phasic shifts in neuronal excitability also create a periodicity in activity levels over time as well as determining absolute levels at any given moment. Our findings suggest that this periodicity in activity can influence the processing of multiple stimulus events stretched over time and the subsequent perception of their timing. This periodicity of neuronal activity may have effects which are independent of alpha amplitude when the stimuli in question are above threshold levels for detection. Alpha activity has been shown to suppress periodically neuronal firing rates in cellular recordings of cat thalamocortical neurons (Lorincz et al., 2009) and in models of thalamic alpha activity (Vijayan and Kopell, 2012). The proposal that alpha phase creates a periodicity in neuronal activity is therefore biologically plausible, and one possible interpretation is that alpha phase reflects a fundamental periodicity in thalamocortical information transfer. This is consistent with the hypothesis that cortical alpha activity is critically influenced by activity within thalamic nuclei which are thought to gate thalamocortical and cortico-cortical transfer (e.g. Hughes and Crunelli, 2005, Lorincz et al., 2009, Saalmann et al., 2012). In contrast, changes in alpha amplitude may serve a distinct function. For example, increased alpha amplitude over task-irrelevant sensory regions may reflect a mechanism which improves detection of high priority unattended inputs which may be salient or threatening. This is predicated on the assumption that certain phases of high amplitude activity promote improved signal to noise ratios with a sampling periodicity of around 10 Hz.

Our findings lend support to the hypothesis that the efficiency of perceptual processes fluctuates periodically and that this is associated with oscillations in the EEG (e.g. Lorincz et al., 2009, Schroeder and Lakatos, 2009, VanRullen and Koch, 2003, Varela et al., 1981). On the basis of their findings, Varela et al. (1981) further suggested that the alpha cycle might relate to a discrete temporal frame during which temporal information is bound and across which it is parsed. This theory of discrete perception was notably proposed by Stroud (1956), although the idea has even earlier antecedents in the literature (e.g. Pitts and McCulloch, 1947, Wiener, 1948). As mentioned above, there is some evidence for the temporal framing of neuronal firing rates (Lorincz et al., 2009), and this hypothesis is also compatible with the finding that the time-course of phase changes influence perception independent of amplitude changes (see also Cravo et al., 2015). The results presented here are in principle compatible with this hypothesis. However, the temporal frame hypothesis is inconsistent with the relatively small variation in detection rates that we observed in this study (see also Gho and Varela, 1988). Similarly, it is inconsistent with the fact that effects of pre-stimulus phase on perceptual performance have only been shown in relation to near-threshold stimuli (e.g. Rice and Hagstrom, 1989, Busch et al., 2009, Mathewson et al., 2009, Neuling et al., 2012). Were phasic fluctuations in neuronal excitability responsible for the strict quantization of sensory input then one might expect them to play a more decisive role in perceptual outcomes that is evident regardless of stimulus properties (e.g. Patterson, 1990). Alternatively, an excitability account of alpha phase might be sufficient to account for the results without recourse to the hypothesis of a temporal frame. Phases associated with raised excitability at onset of the first target would promote improved processing regarding both the targets and the SOA, especially when the SOA is around perceptual threshold. Rather than proposing that all stimuli that occur within an excitable phase period are temporally bound, this account instead suggests that they would be more likely to be perceived correctly as asynchronous. In contrast, less excitable phases may be related to a relatively worse quality of processing and a less clear perceptual experience for judgements near threshold. Fluctuations in neuronal excitability over time would therefore admit periodic fluctuations in perceptual outcome, even for simultaneity judgements, without committing to discrete temporal frames of perception. Critically, this suggestion allows for alpha phase to exert a modulatory influence on perception rather than a decisive one. This is in turn compatible with the relatively modest influence phase was shown to have on the proportion of asynchronous judgements, as well its demonstration only with regard to threshold-level stimuli. It is not possible, however, to differentiate between these alternate explanations on the basis of our findings. This experiment therefore presents evidence consistent with the idea of periodic fluctuations in processing and perception. Although possible, it is not at present clear whether these periodic fluctuations necessarily form the limit of temporal resolution or represent a dedicated quantization of sensory input over time as has been previously suggested (Varela et al., 1981).

Since phase modulates the detection of near-threshold visual stimuli (Busch et al., 2009, Mathewson et al., 2009) it is possible that certain phases delayed detection of the leading target so that it was perceived closer in time to the second. Phase might therefore have biased simultaneity judgements via its influence on stimulus detection rather than its direct influence on temporal perception. However, the SOA, rather than target-visibility, was kept at perceptual threshold, and there is no evidence that alpha phase influences visual detection for supra-threshold stimuli. We therefore suggest that phase influenced the perception of relative timing as opposed to stimulus detection per se.

The left hemisphere topography observed in this study is inconsistent with previous findings of bilateral attentional modulation of alpha amplitude (e.g. Sauseng et al., 2005, Worden et al., 2000). The analysis of attentional modulation in each hemisphere separately confirmed that the effect demonstrated here was indeed restricted to the left hemisphere, and this topography may reflect the temporal nature of this task which differs from previous studies that focused on stimulus detection paradigms. Interestingly, this is not the first study to report a left hemisphere bias for tasks involving temporal perception (Carmon and Nachshon, 1971, Efron, 1963, Hernandez et al., 2015). The particular importance of the left hemisphere for motor-sequencing (e.g. Harrington and Haaland, 1992) and language processing (e.g. Frost, 1999), both of which require the processing of fine temporal sequences, might explain why temporal perception of small intervals would preferentially engage the left hemisphere (Hernandez et al., 2015), although such a conclusion requires more dedicated testing. It also remains an intriguing possibility that a study demonstrating a bilateral attentional suppression of alpha amplitude might also report a bilateral alpha phase effect and this represents a future area of interest.

Further constraints of the effect were demonstrated in the wider analysis of all electrodes that was conducted after the primary analyses restricted to the left posterior cluster revealed no interaction between alpha phase and amplitude. As with the modest influence that phase was shown to have on the proportion of asynchronous judgements, this wider analysis suggests that the effect of phase is relatively modest. That it did not survive the noise associated with unconstrained analyses may explain subsequent failures to replicate the original finding (see VanRullen and Koch, 2003). That the effect was only seen in an analysis targeted at electrodes exhibiting an attentional suppression of alpha amplitude suggests that it may be important to focus analyses on regions where the alpha rhythm is demonstrably responsive to task demands. By triggering stimuli in response to real-time changes in alpha phase, the original finding by Varela et al. (1981) was similarly targeted at a limited number of electrodes (PZ, O1 and O2) and such approaches are likely to be more sensitive. Given that the effect size is modest, future investigation might benefit from focused hypotheses and we draw attention to the fact that both our analysis and that of Varela demonstrated an effect at occipital site O1. Indeed, the localisation of effects to posterior sensory regions might be expected on account of the visual nature of the stimuli, and Varela and colleagues explicitly reported a stronger relationship between alpha phase and judgements of simultaneity at occipital over parietal sites (Varela et al., 1981, pg. 680). As the current study was interested in sites demonstrating an attentional modulation of alpha amplitude we had no a priori reason to localize analysis to occipital electrodes. However, on the basis of this study and the original (Varela et al., 1981) future research may have some justification for a priori targeting of occipital sites, especially O1.

This study provides renewed evidence that changes in pre-stimulus alpha phase can influence temporal perception. We therefore add to the rich literature suggesting that alpha phase indexes functionally important changes in neuronal activity. The results are consistent with the hypothesis that alpha phase reflects fluctuations in neuronal excitability that can periodically modulate perceptual processes.
